# Efficiencies Evaluation of Photocatalytic Paints Under Indoor and Outdoor Air Conditions

**DOI:** 10.3389/fchem.2020.551710

**Published:** 2020-10-23

**Authors:** Federico Salvadores, Martin Reli, Orlando M. Alfano, Kamila Kočí, María de los Milagros Ballari

**Affiliations:** ^1^Instituto de Desarrollo Tecnológico para la Industria Química (Universidad Nacional del Litoral and Consejo Nacional de Investigaciones Científicas y Técnicas), Santa Fe, Argentina; ^2^Institute of Environmental Technology, Vysoká Škola Báňská—Technical University of Ostrava, Ostrava, Czechia

**Keywords:** air decontamination, modified TiO_2_, photocatalytic paints, photonic efficiency, quantum efficiency, ultraviolet light, visible light

## Abstract

The removal of indoor and outdoor air pollutants is crucial to prevent environmental and health issues. Photocatalytic building materials are an energy-sustainable technology that can completely oxidize pollutants, improving *in situ* the air quality of contaminated sites. In this work, different photoactive TiO_2_ catalysts (anatase or modified anatase) and amounts were used to formulate photocatalytic paints in replacement of the normally used TiO_2_ (rutile) pigment. These paints were tested in two different experimental systems simulating indoor and outdoor environments. In one, indoor illumination conditions were used in the photoreactor for the oxidation of acetaldehyde achieving conversions between 37 and 55%. The other sets of experiments were performed under simulated outdoor radiation for the degradation of nitric oxide, resulting in conversions between 13 and 35%. This wide range of conversions made it difficult to directly compare the paints. Thus, absorption, photonic, and quantum efficiencies were calculated to account for the paints photocatalytic performance. It was found that the formulations containing carbon-doped TiO_2_ presented the best efficiencies. The paint with the maximum amount of this photocatalyst showed the highest absorption and photonic efficiencies. On the other hand, the paint with the lowest amount of carbon-doped TiO_2_ presented the highest value of quantum efficiency, thus becoming the optimal formulation in terms of energy use.

## Introduction

The air quality can affect people's health and the environment in different means (Fiore et al., 2015). The short-term effects of the exposure to polluted air include irritation of the eyes, nose, and throat, headaches, nausea, and allergic reactions. Among the long-term effects, respiratory syndromes, heart disease, and cancer can be mentioned (Guillerm and Cesari, 2015). The air of an indoor environment can be polluted basically in two ways: (i) the pollutant is generated or released inside the room and accumulated due to a poor ventilation and (ii) the outside generated contaminant that enters the room through open doors or windows and the ventilation system. According to the World Health Organization (WHO), household air pollution is the cause of 3.8 million premature deaths annually (WHO website Air pollution, 2019). Outdoor air pollution is not less hazardous, being the main environmental risk to health also according to the WHO and contributing to climate change, too.

Some examples of air pollutants are nitrogen oxides (NO_*x*_), sulfur dioxide (SO_2_), and volatile organic compounds (VOCs). The term nitrogen oxides (NO_*x*_) includes nitric oxide (NO) and nitrogen dioxide (NO_2_) among other highly reactive gases. They are mainly formed in urban areas through combustion processes in vehicles, power plants, and other industrial sources and can cause photochemical smog and acid rain, which contribute to global warming (Fiore et al., 2015). Acetaldehyde is a recurrent VOC present in indoor environments that can also be formed during combustion processes and emitted by different sources in homes like building materials, hardwood, plywood, laminate floorings, adhesives, paints, and varnishes (Missia et al., 2010). It can cause eyes, skin, and respiratory tract irritation, and it is classified as a probable carcinogen. Because of their negative effects, these outdoor and indoor air pollutants must be removed before their concentrations reach harmful levels.

Heterogeneous photocatalytic oxidation (PCO) is a well-known method to remove air and water contaminants (Ibhadon and Fitzpatrick, 2013). Recently, novel nanostructured semiconductors were studied as photocatalysts for environmental remediation, like CeO_2_ and cerium-doped photocatalysts (Liu et al., 2013; Muñoz-Batista et al., 2015; Montini et al., 2016; Šihor et al., 2017), graphitic carbon nitride (g-C_3_N_4_) (Baca et al., 2019), graphene oxide composites (Gupta et al., 2015; Han et al., 2016; Li et al., 2018), silver nanoparticles (AgNPs) (Yola et al., 2013), ternary ZnO/Ag/Mn_2_O_3_ composite (Saravanan et al., 2015), AgI/WO_3_ heterojunctions (Wang et al., 2016), and SnO_2_ nanoparticles (Elango and Roopan, 2016). However, anatase titanium dioxide (TiO_2_) is still the most extensively investigated semiconductor due to its non-toxicity, high stability, and good cost/efficiency relation (Hoffmann et al., 1995; Malato et al., 2009; Muñoz-Batista et al., 2019). Titanium dioxide can normally be activated under ultraviolet (UV) light, i.e., 200–400 nm, although it can be used in the visible spectrum after some modifications like dye sensitization, doping with transition metals, or with non-metal anions (Daghrir et al., 2013; Banerjee et al., 2014; Khaki et al., 2017).

One of the emerging applications of this technology is the combination of TiO_2_ with construction materials obtaining self-cleaning surfaces with air-purifying capacity (Fujishima et al., 1999; Chen and Poon, 2009; Ballari and Brouwers, 2013). Diverse publications have analyzed photocatalytic paints for the degradation of different air pollutant models: first is the NO_*x*_ in the form of NO (Maggos et al., 2007a,b; Águia et al., 2010, 2011a,b; Laufs et al., 2010; Ângelo et al., 2014) or NO_2_ (Maggos et al., 2007a,b; Salthammer and Fuhrmann, 2007; Laufs et al., 2010; Gandolfo et al., 2015, 2017). Some of these works have followed the standard ISO 1524 (2007) using a flat plate continuous photoreactor irradiated with UV light; other authors have employed reaction chambers (Maggos et al., 2007a,b; Salthammer and Fuhrmann, 2007) or other type of flow reactors (Laufs et al., 2010; Ângelo et al., 2014) and have used sunlight (Ângelo et al., 2014) or visible light (Salthammer and Fuhrmann, 2007) as the energy source for the NO_*x*_ photocatalytic oxidation. Second are VOCs such as formaldehyde (Salthammer and Fuhrmann, 2007; Fu et al., 2013), acetaldehyde (Salvadores et al., 2020a,b), n-decane (Monteiro et al., 2014), perchloroethylene (Monteiro et al., 2015), and toluene (Maggos et al., 2007c) applying UV radiation in most of them. Third are other toxic or dangerous compounds like CO (Salthammer and Fuhrmann, 2007) and benzo-[a]-pyrene (Tryba et al., 2014). In the latter investigation, visible radiation was applied in addition to UV light. Some of these studies were carried out with commercially available or manufacturer-produced photocatalytic paints (Tryba et al., 2014; Gandolfo et al., 2015). On the other hand, other works were focused on their own formulations and have studied the influence of the paint components and the type and amount of TiO_2_ on the contaminant degradation (Águia et al., 2010, 2011a,b).

From the literature review of photocatalytic paints and coatings, it can be seen that very wide results on the contaminant degradation and selectivity were obtained. This large variability in results comes principally from the employment of different photoreactor configurations and sizes, operating conditions, tested pollutants, photocatalytic materials, and paint compositions. Thus, the direct comparison between all these systems is not appropriate, at least by evaluating only the pollutant conversion. A way of becoming independent, to a certain extent, from the operating conditions and experimental configurations, such as catalytic area, air flow, contaminant concentration, and radiation flux, is the calculation of quantum and photonic efficiencies (QE and PE, respectively) of the photocatalytic reacting system (Imoberdorf et al., 2007; Passalía et al., 2013; Muñoz-Batista et al., 2014).

In this work, undoped and carbon-doped TiO_2_ in different amounts were used in the formulation of water-based paints and pseudo-paints. Water-based paints were chosen over other formulations because they release significantly fewer VOCs during the drying process and, therefore, are more environment friendly. In a previous work (Salvadores et al., 2020b), the carbon-doped TiO_2_ paints were applied for an intrinsic kinetic study of acetaldehyde degradation applying indoor illumination. In this new contribution, the photocatalytic paint coatings were tested for two typical situations: (i) under visible light source for the degradation of a typical indoor air contaminant (acetaldehyde) and (ii) under UV radiation for the degradation of a common outdoor air pollutant (NO_*x*_). The optical properties of the paints were measured, and the local superficial rate of photon absorption (LSRPA) was calculated to correlate them with the removal capability of the pollutant and to evaluate the coatings performance in terms of the photonic and quantum efficiencies. In addition, the photocatalytic activity after a long-term reaction under UV radiation of the designed materials was assessed.

## Materials and Methods

### Paints and Pseudo-Paints Preparation and Coatings Application

Diverse paints and pseudo-paints were formulated varying the TiO_2_ types, all commercially available, and its amount. Table 1 presents the formulated paints and pseudo-paints and the type and amount of TiO_2_ employed. The photocatalytic anatase TiO_2_ powders were (i) KRONOClean 7050 undoped TiO_2_ (TiO_2_-Und), which has a specific surface area (S_*BET*_) of 341 m^2^/g (Patzsch and Bloh, 2018) and an average particle size (APS) of 15 nm, and (ii) KRONOClean 7000 carbon-doped TiO_2_ (TiO_2_-C) that presents an S_*BET*_ of 251 m^2^/g and APS of 15 nm. The KRONOClean 7000 was thoroughly characterized by Arimi et al. (2019) and Tobaldi et al. (2015). The carbon doping was carried out through an aromatic carbon-based sensitizer layer on the photocatalyst (Arimi et al., 2019). The KRONOClean 7050 powder is a pristine TiO_2_, being in this work a reference catalyst for the carbon-doped TiO_2_ performance. The reported S_*BET*_ and APS of KRONOClean 7000 and the APS of KRONOClean 7050 were provided by the manufacturer. In addition, a photocatalytic paint for comparison purposes was formulated using the benchmark Aeroxide® P25 (TiO_2_-P25), which is mainly a mixture of anatase and rutile titanium dioxide phases (Jiang et al., 2018). Finally, non-photocatalytic KRONOS 2360 TiO_2_ in rutile crystalline form (TiO_2_-Rut) was used as the blank sample for the photocatalytic reaction. The content of TiO_2_ is larger than 92% according to the manufacturer.

**Table 1:** Characteristics and deposited amounts of paints and pseudo-paints.

**TiO_**2**_ type/brand name**	**Paint/pseudo-paint**	**TiO_**2**_ amount (% w/w)**	**CaCO_**3**_ amount (% w/w)**	**Fineness of grind (μm)**	**Specific load of dry paint** **× 10**^****3****^ **(g/cm**^****2****^**)**	
					**Indoor-like experiments**	**Outdoor-like experiments**
Undoped anatase/KRONOClean 7050	18TiO_2_-Und	18	18	20	1.10	1.59
	PP-TiO_2_-Und	22	–	18	0.71	–
Carbon-doped anatase/KRONOClean 7000	18TiO_2_-C	18	18	21	1.18	1.13
	14TiO_2_-C	14	22	24	0.89	1.35
	12TiO_2_-C	12	24	22	0.91	1.63
	PP-TiO_2_-C	22	–	17	0.64	–
	13CaCO_3_-18TiO_2_-C	18	13	21	1.08	–
	8CaCO_3_-18TiO_2_-C	18	8	22	1.02	–
Mixture of anatase and rutile/Aeroxide® P25	18TiO_2_-P25	18	18	24	1.41	–
Rutile/KRONOS 2360	18TiO_2_-Rut	18	18	16	1.01	0.78
–	PP-noTiO_2_	–	22	18	–	–

The paint formulation used was 30% w/w of distilled water, 33.4% w/w of resin BASF ACRONAL RS 723, 0.6% w/w of dispersing agent BASF DISPEX AA 4146, and the total amount of solids, i.e., pigment (TiO_2_) and extender (CaCO_3_ Cicarelli, >99% purity), was maintained constant in 36% w/w. The maximum amount of TiO_2_ in paints was 18% w/w (in paints 18TiO_2_-Und, 18TiO_2_-C, 18TiO_2_-P25, and 18TiO_2_-Rut). In addition, other paints were elaborated varying the carbon-doped TiO_2_ amount to 14 and 12% w/w (paints 14TiO_2_-C and 12TiO_2_-C, respectively).

On the other hand, two paints were formulated varying the amount of CaCO_3_, while the percentage of carbon-doped TiO_2_ remained constant in 18% w/w (paints 13CaCO_3_-18TiO_2_-C and 8CaCO_3_-18TiO_2_-C). Finally, different pseudo-paints were elaborated, omitting one component of the solid matrix, i.e., the extender (PP-TiO_2_-Und, and PP-TiO_2_-C) or the TiO_2_ (PP-noTiO_2_) but maintaining the original weight of the other components of the equivalent paint.

For the paints elaboration, TiO_2_ and the CaCO_3_ were first hand milled and dried at 110°C. Then, these solids were incorporated to a solution of distilled water and dispersing agent while mixing at 300 rpm. The resin was added in the final step to complete the paint.

The paints were applied with an aerograph on acrylic plates of ~20 × 9.4 cm for the acetaldehyde degradation and 9.4 × 4.8 cm for the NO_*x*_ experiments. After the paint deposition on each side, the plates were dried at ambient humidity and temperature for 24 h. Table 1 also shows the final amounts of deposited dry paint per unit of surface area for both indoor and outdoor reacting systems.

The photocatalytic coatings were exposed to visible radiation lamps previous to the air decontamination experiments. The resin covering the TiO_2_ particles was degraded with this procedure, thus allowing the interaction between the photocatalyst and the contaminated air (Marolt et al., 2011).

### Characterization of the Paints and Pseudo-Paints

The fineness of grind of the fluid paints and pseudo-paints was determined in a grindometer (SCHWYZ GRIN210-1) with a measurement range from 0 to 25 μm. This measurement range corresponds to the dispersion fineness of pigment-vehicle systems (ISO 22197-2, 2013).

Images of the elaborated coatings were taken with a scanning electron microscope (SEM JEOL JSM-35C) and a transmission electron microscope (TEM JEOL JEM-2100 Plus).

X-ray photoelectron spectroscopy (XPS) was used to determine superficial titanium and oxygen on three different paint coatings. This analysis was performed in a Specs Multitechnique instrument with dual X-ray source Mg/Al, PHOIBOS 150 hemispherical analyzer operating in fixed analyzer transmission (FAT) mode, step energy of 30 eV, Mg anode at 100 W, and pressure <2 × 10^−9^ mbar. The analyzed bands were 2p for the Ti and 1s for O and for C. The reference was C 1s, and the spectra were corrected at 284.6 eV.

In addition, the spectral diffuse reflectance and transmittance of the coated acrylic plates were determined between wavelengths (λ) of 300 and 800 nm in a spectroradiometer (Optronic OL Series 750) equipped with an OL 740–70 integrating sphere reflectance attachment. Based on these optical properties, the fraction of energy absorbed by the paint film was calculated according to the methodology reported elsewhere (Ballari et al., 2016).

### Indoor-Like Photocatalytic Experiments

**Figure 1:**
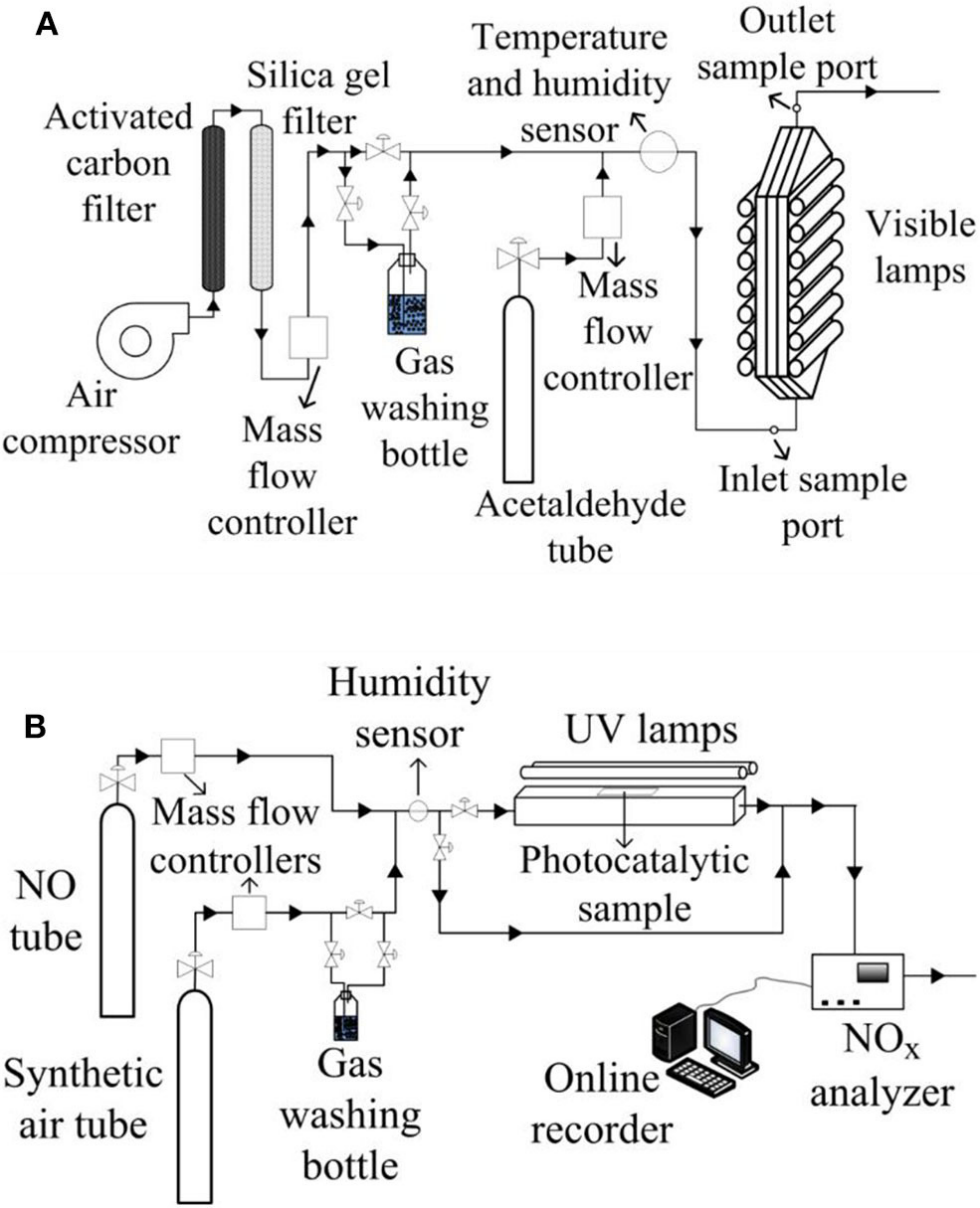
Experimental setups to carry out both contaminants photocatalytic degradation for: **(A)** indoor like experiments, **(B)** outdoor like experiments.

The experimental device to carry out the acetaldehyde oxidation consists of a continuous flat plate photoreactor with the acrylic plate coated with the photocatalytic paint (Figure 1A). Acetaldehyde gas, stabilized in nitrogen (certified 300 ppm, Praxair) and mixed with air to reach the desired inlet concentration (5 ppm), was fed into the photocatalytic reactor. Mass controllers were used to set the flowrates. A fraction of the air flow passed through a gas washing bottle with the purpose of adjusting the humidity level, which was measured with a thermohygrometer (HDT HygroTherm 6004). The gas mixture was divided into two streams at the reactor inlet, flowing between the reactor walls and the acrylic plate. The photoreactor was irradiated on both sides with fluorescent visible light lamps (λ = 310–710 nm). The radiation flux was measured with a radiometer (IL1700) with SED#0339470 detector and an F#29411 filter. The spectral emission of the lamp was measured with a spectrometer Ocean Optics USB2000+UV-VIS-ES (Supplementary Figure 1 of the Supplementary Material). A gas chromatograph with an FID detector (HP Series II 5890) was used to determine the contaminant and intermediates concentrations at the reactor inlet and outlet by performing a direct injection of the gas sample. Table 2 presents the experimental setup characteristics and operating conditions for the indoor-like experiments. Even though this reactor dimensions do not follow the ISO 22197-1 (2011) for acetaldehyde degradation, some adopted operating conditions were the proposed ones by this standard (pollutant inlet concentration, relative humidity, and flowrate).

**Table 2:** Experimental setup and operating conditions.

	**Indoor-like experiments**	**Outdoor-like experiments**
Reactor (length × width)	20 cm × 10 cm	10 cm × 5 cm
Reactor thickness	0.2 cm each side	0.5 cm
Residence time	4.8 s	0.5 s
Lamp brand/model–input power	GE F4T5/CW−4 W	OSRAM EVERSUN L40/79K−40 W
Type of light	Visible daylight	UV
Number of lamps	7 on each side of the photoreactor	2 on one side of the photoreactor
Emission wavelength	310–710 nm	310–410 nm
Photocatalytic paint sample (length × width)	20 cm × 9.4 cm	9.4 cm × 4.9 cm
Irradiated surface, *Area*	376 cm^2^	46.06 cm^2^
Flowrate, *Q*	16.7 cm^3^/s	50.0 cm^3^/s
Relative humidity	50%	50%
Incident Radiation Flux, *q*_*w*_	2.36 × 10^−8^ Einstein/cm^2^/s (58.8 W/m^2^) each side	3.55 × 10^−9^ Einstein/cm^2^/s (10 W/m^2^)
Model pollutant	Acetaldehyde	Nitric oxide
Inlet pollutant concentration, *C*_*y,in*_	2.05 × 10^−10^ mol/cm^3^ (5 ppm)	4.09 × 10^−11^ mol/cm^3^ (1 ppm)

### Outdoor-Like Photocatalytic Experiments

The photoreactor used for the NO_*x*_ experiments (Figure 1B) was built in accordance with the ISO method 22197-1 (2007). The reactor was fed by NO gas (certified 300 ppm stabilized in N_2_, SIAD) mixed with synthetic air (final concentration of NO was 1 ppm) and irradiated from the top by two UV lamps (λ_*peak*_ = 365 nm). To set the flowrate of air and NO, mass controllers were used. To adjust the humidity level, a fraction of the air flow was bypassed through a gas washing bottle. The photoreactor was provided with a non-irradiated entrance to develop the gas flow. The radiation flux was measured with an optical radiometer UVP MS-100 with an MS-136 sensor, and the spectral emission of the UV lamps was provided by the supplier (Supplementary Figure 1 of the Supplementary Material). The outlet contaminant concentration was analyzed by an online chemiluminescence NO_*x*_ analyzer (Ecotech EC9841). The employed operating conditions and principal characteristic of the experimental setup can be found in Table 2.

For this reacting system, five consecutive tests were done with the same paint formulation sample and experimental conditions in order to assess the aging and activity loss of the photocatalytic material. So as to release possible reaction intermediates adsorbed on the surface, vacuum was applied to the paint samples between the tests 4 and 5. Liquid tests of the adsorbed intermediates were not performed due to not having the possibility to conduct the analysis.

## Results and Discussion

### Analysis of the Paints Characteristics

The results of the fineness of grind measurements of the paints and pseudo-paints are summarized in Table 1. The photocatalytic paints present an average of grinding fineness of 22.4 ± 2.1 μm, while the pseudo-paints with only TiO_2_ show an average of 18.3 ± 1.3 μm. This could be an indication that the CaCO_3_ and the TiO_2_ in the photocatalytic paints form bigger particle conglomerates than the TiO_2_ alone in the pseudo-paints. However, the paint 18TiO_2_-Rut presents a grind fineness lower than the pseudo-paints' average granulometry. A possible reason for this is that the rutile TiO_2_ is specially designed for paint production and, consequently, with a better dispersion obtained during the paint elaboration.

**Figure 2:**
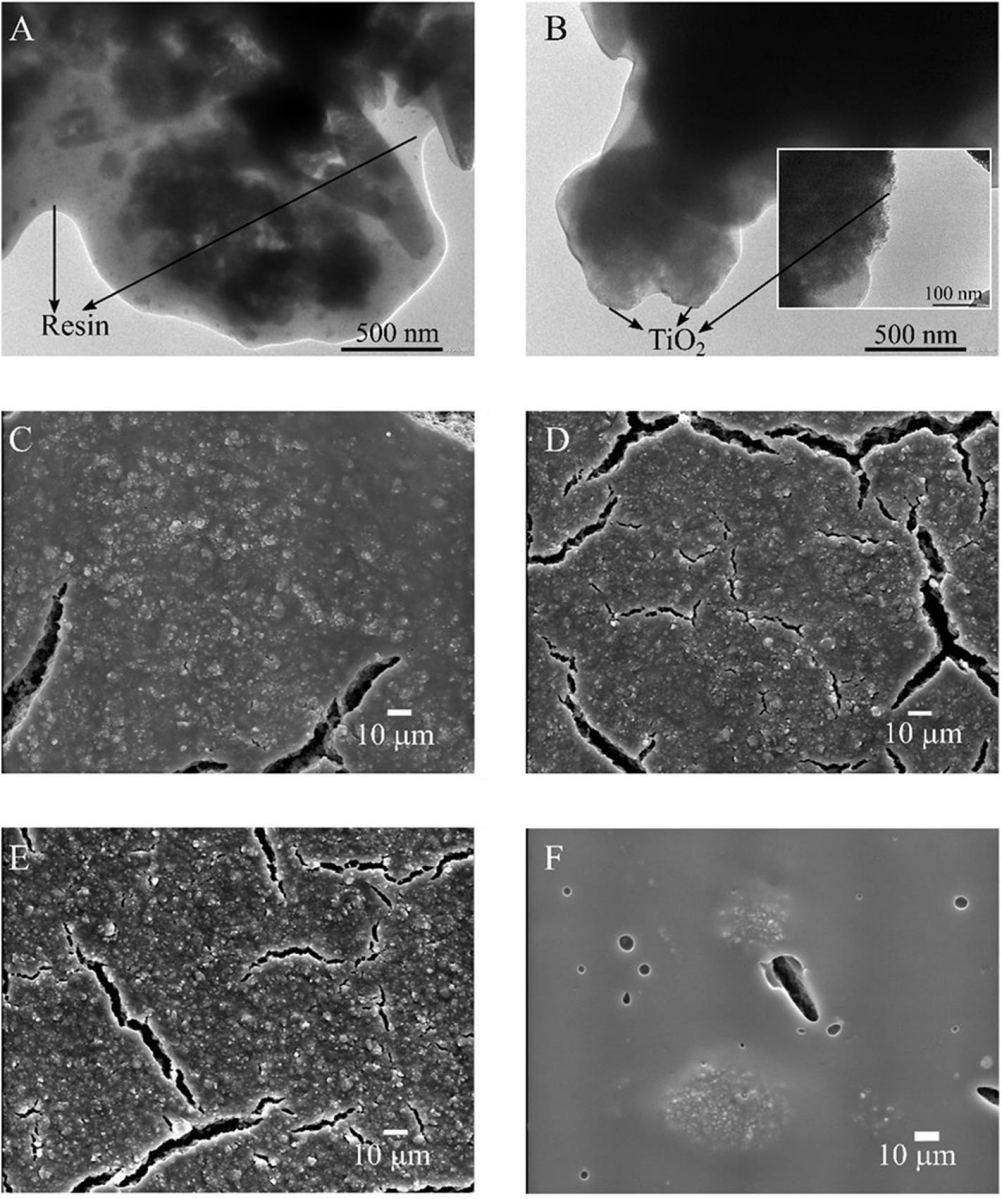
Micrographs of paints coatings: **(A)** TEM 18TiO_2_-C before being irradiated, **(B)** TEM 18TiO_2_-C after being irradiated, **(C)** SEM 18TiO_2_-C, **(D)** SEM 14TiO_2_-C, **(E)** SEM 12TiO_2_-C and **(F)** SEM 18TiO_2_-Und.

Figure 2 presents TEM and SEM images of the different paint coatings that have been treated previously under illumination, with the exception of the sample shown in Figure 2A, which was not irradiated. In the TEM images, it can be seen that the particles in the non-treated paint film (Figure 2A) are less exposed to the surface than the one previously irradiated (Figure 2B). This is an indication that TiO_2_ particles protrude after the irradiation curing procedure due to surface resin degradation (Marolt et al., 2011). The last claim is also supported by X-ray photoelectron spectroscopy (XPS) analysis of 18TiO_2_-C paint coating that revels an increase in the surface Ti/O ratio from 0.011 to 0.015 after the irradiation curing (see Supplementary Table 2 and Supplementary Figure 3 of the Supplementary Material). It has to be noticed that degradation of the paint after more than 140 h of reaction was not observed (Salvadores et al., 2020a). Nevertheless, durability test for longer periods in indoor and outdoor like conditions has to be evaluated in forthcoming experiments.

On the other hand, the SEM images (Figures 2C–F) show the coatings in a larger scale after being irradiated. Paints 18TiO_2_-C, 14TiO_2_-C, and 12TiO_2_-C (Figures 2C–E) are very similar because they contain the same photocatalyst; however, as the TiO_2_ quantity diminishes, the formation of microcracks is more noticeable. In addition, for the paints with TiO_2_-C, the Ti/O ratio at the coating surface diminishes from 0.019 to 0.015 when the photocatalyst amount increases from 12 to 18% w/w (XPS measurements, Supplementary Table 2 and Supplementary Figure 3). This suggests that a lower amount of TiO_2_-C results in smaller and better distributed particles agglomerations on the surface (Salvadores et al., 2020b). Finally, it can be observed that the paint 18TiO_2_-Und (Figure 2F) shows a very smooth matrix and presents bigger particles agglomerations despite that the fluid paint has a fineness of grind similar to the 18TiO_2_-C paint (Table 1). This phenomenon could be related to the agglomeration mechanism and stability in polymeric matrix.

### Optical Properties of the Photocatalytic Paint Coatings

The absorbed radiation fraction of the TiO_2_ inside the paint matrix was calculated according to a methodology based on a radiative flux balance in a three-layer system (Ballari et al., 2016), using experimental measurements of the diffuse reflectance and transmittance of the acrylic plate and the coated acrylic on both sides (Supplementary Figure 2 of the Supplementary Material). The spectral fraction of reflected radiation from the paint film deposited on the acrylic plate can be calculated as:

(1) $$\begin{array}{l} R_{paint,\lambda }=\frac{\left(R_{paint,acr,paint,\lambda }T_{acr,\lambda }-T_{paint,acr,paint,\lambda }R_{acr,\lambda }\right)}{\left(T_{paint,acr,paint,\lambda }T_{acr,\lambda }^{2}-T_{paint,acr,paint,\lambda }R_{acr,\lambda }^{2}+T_{acr,\lambda }\right)}\; \end{array}$$

where *R* and *T* denote the diffuse reflectance and diffuse transmittance, respectively, the subscript “paint, acr, paint” indicates the system formed by the acrylic plate and the paint deposited on both sides of it, and the subscript “acr” denotes the unpainted acrylic plate.

The transmittance of the paint film deposited on the acrylic plate for each wavelength is:

(2) $$\begin{array}{l} T_{paint,\lambda }=\sqrt{\frac{\left(R_{paint,acr,paint,\lambda }-R_{paint,\lambda }\right)\left\{1-R_{paint,\lambda }\left[R_{acr,\lambda }+\frac{T_{acr,\lambda }^{2}R_{paint,\lambda }}{\left(1-R_{acr,\lambda }R_{paint,\lambda }\right)}\right]\right\}}{R_{acr,\lambda }+\frac{T_{acr,\lambda }^{2}R_{paint,\lambda }}{\left(1-R_{acr,\lambda }R_{paint,\lambda }\right)}}} \end{array}$$

Finally, the spectral radiation absorption fraction (*A*) of the paint film deposited on the acrylic plate can be calculated by:

(3) $$\begin{array}{l} A_{paint,\lambda }=1-R_{paint,\lambda }-T_{paint,\lambda } \end{array}$$

Due to the difficulty of depositing the same exact amount of paint for every experiment, the radiation absorption fraction of the different paints was divided by the specific load, i.e., the *Weight* (g) of the deposited paint divided by the acrylic area, *Area* (cm^2^):

(4) $$\begin{array}{l} A_{paint,norm,\lambda }=\frac{A_{paint,\lambda }}{\left(Weight/Area\right)} \end{array}$$

where the subscript “norm” indicates that it is a normalized property.

In order to know the amount of radiation absorbed by the TiO_2_ inside the paint matrix, Equation 5 is proposed:

(5) $$\begin{array}{l} A_{TiO_{2},norm,\lambda }=A_{paint,norm,\lambda }-A_{PP-noTiO_{2},norm,\lambda } \end{array}$$

where *A*_*PP*−*noTiO*_2_,*norm*,λ_ (cm^2^/g) is the normalized radiation absorption fraction of the pseudo-paint that does not contain TiO_2_ in its formulation. The purpose of this is to discount the absorbed radiation by other components of the paint different from the TiO_2_. This methodology is based on the fraction of the radiation absorption of the paint that is the sum of this optical property of each component.

**Figure 3:**
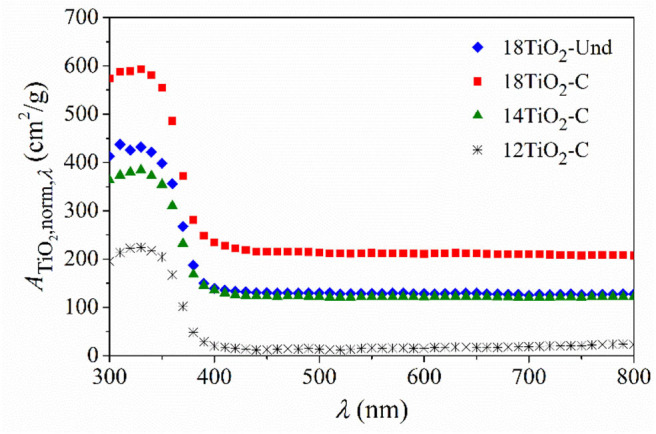
Radiation absorption fraction of the TiO_2_ in the different photocatalytic paints normalized by the deposited specific load of the dried paint.

Figure 3 shows the calculated *A*_*TiO*_2_,*norm*,λ_ as a function of wavelength. Carbon-doped TiO_2_ in paint 18TiO_2_-C has an upper absorption than normal TiO_2_ in paint 18TiO_2_-Und, although the same amount of photocatalyst was used to prepare these two paints, i.e., 18% w/w. When the carbon-doped TiO_2_ amount is decreased in the formulations of paints 14TiO_2_-C and 12TiO_2_-C, the radiation absorption fraction relative to the PP-noTiO_2_ also decreases.

In addition, doped and undoped photocatalysts present similar radiation absorption edge below 400 nm. The calculated optical band gap of carbon-doped TiO_2_ using the Kubelka–Munk methodology is 3.2 eV (data not shown) corresponding to a radiation wavelength of 386 nm. This value is in accordance to the reported band gap energy by the bibliography (Kete et al., 2014; Tobaldi et al., 2015; Sankova et al., 2018) and similar to the undoped TiO_2_ one. In addition, it should be noticed that similar absorbance in the visible spectrum range for KRONOClean 7050 and other laboratory-synthetized anatase TiO_2_ was reported elsewhere (Kalaivani and Anilkumar, 2018; Shaitanov et al., 2018).

### Typical Photocatalytic Tests

From the acetaldehyde photocatalytic oxidation mechanism proposed in the literature, the following sequence of stable intermediates can be expected (Sauer and Ollis, 1996; Ye et al., 2006):

$$\begin{array}{l} \text{Acetaldehyde}\to \text{Formaldehyde}\to \text{Formic Acid}\\ \;\to \text{Carbon Dioxide} \end{array}$$

During the acetaldehyde degradation experiments under indoor conditions, only formaldehyde at low concentrations was detected as the photoreaction intermediate (Salvadores et al., 2016). On the other hand, no formic acid was detected within the detection limits (0.05 ppm), which can be an indication of the good degradation performance of the paints under visible light lamps.

**Figure 4:**
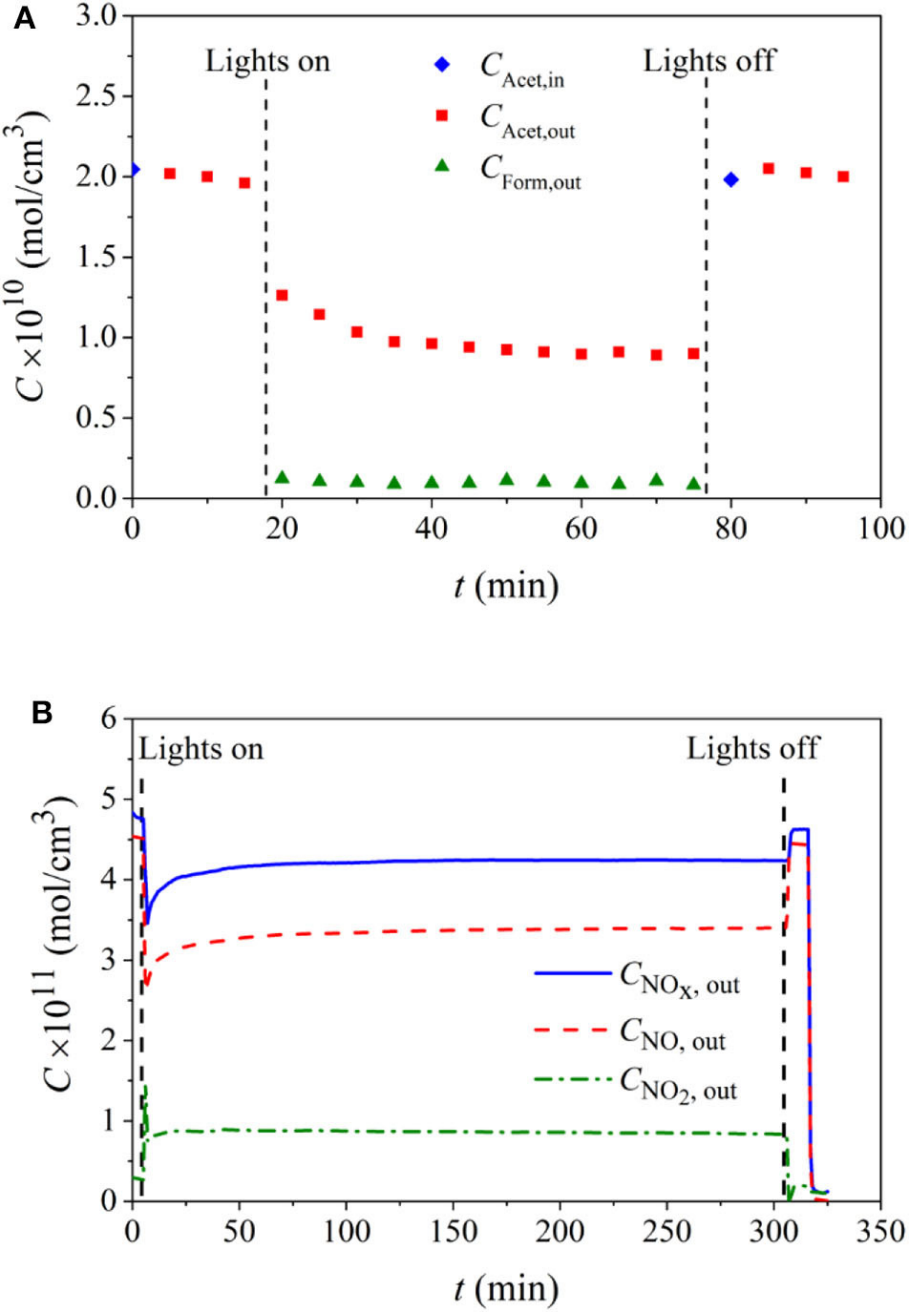
Contaminant and reaction intermediates concentration evolution for paint 18TiO_2_-C formulation and for the degradation of: **(A)** acetaldehyde under visible light, **(B)** NO under UV light.

Figure 4A shows the concentration evolution of the pollutant and the main intermediate during an experimental run applying paint 18TiO_2_-C.

On the other hand, the commonly accepted reaction pathway for the photocatalytic degradation of nitric oxide is (Ballari et al., 2010; Šihor et al., 2017):

$$\begin{array}{l} \text{NO}\to {\text{NO}}_{2}\to \;{\text{HNO}}_{3} \end{array}$$

For the system investigated here, NO_2_ generation was observed as a reaction intermediate. Figure 4B shows an example of an experimental run employing also paint 18TiO_2_-C, in which the NO_*x*_ concentration is the sum of NO and NO_2_ concentrations.

### Air Contaminants Conversion

The contaminants conversion *X*_*y*_ (%) was calculated as:

(6) $$\begin{array}{l} X_{y}=\frac{\left(C_{y,in}-C_{y,out}\right)}{C_{y,in}}\times 100 \end{array}$$

where the subscript “y” denotes the model pollutant, i.e., acetaldehyde or NO, *C*_*y,in*_ (mol/cm^3^) is the inlet concentration of the pollutant, and *C*_*y,out*_ (mol/cm^3^) corresponds to the outlet concentration at the end of each experiment. The acetaldehyde outlet concentration was an average of the last four samples operating in steady state after 60 min of reaction for the indoor-like experiments. On the other hand, for the outdoor-like experiments, the last 20 samples in steady state after 300 min of reaction were used for the calculation of the NO average outlet concentration. The statistical analysis was conducted based on Student's t-distribution for small samples size to calculate the 95% confidence interval of the average conversions. In addition, a reproducibility test was done preparing three coatings with the paint of 18TiO_2_-C, finding an average acetaldehyde conversion of 55.3 ± 1.6%. In addition, the global conversion *X*_*y,g*_ (%) was calculated taking into account the reaction intermediates formation:

(7) $$\begin{array}{l} X_{g,y}=\frac{\left(C_{y,in}-C_{y,out}-C_{z,out}\right)}{C_{y,in}}\times 100 \end{array}$$

where “z” represents the secondary pollutant that is formed during the photoreaction, i.e., formaldehyde for the indoor-like experiments and NO_2_ for the outdoor-like experiments.

**Figure 5:**
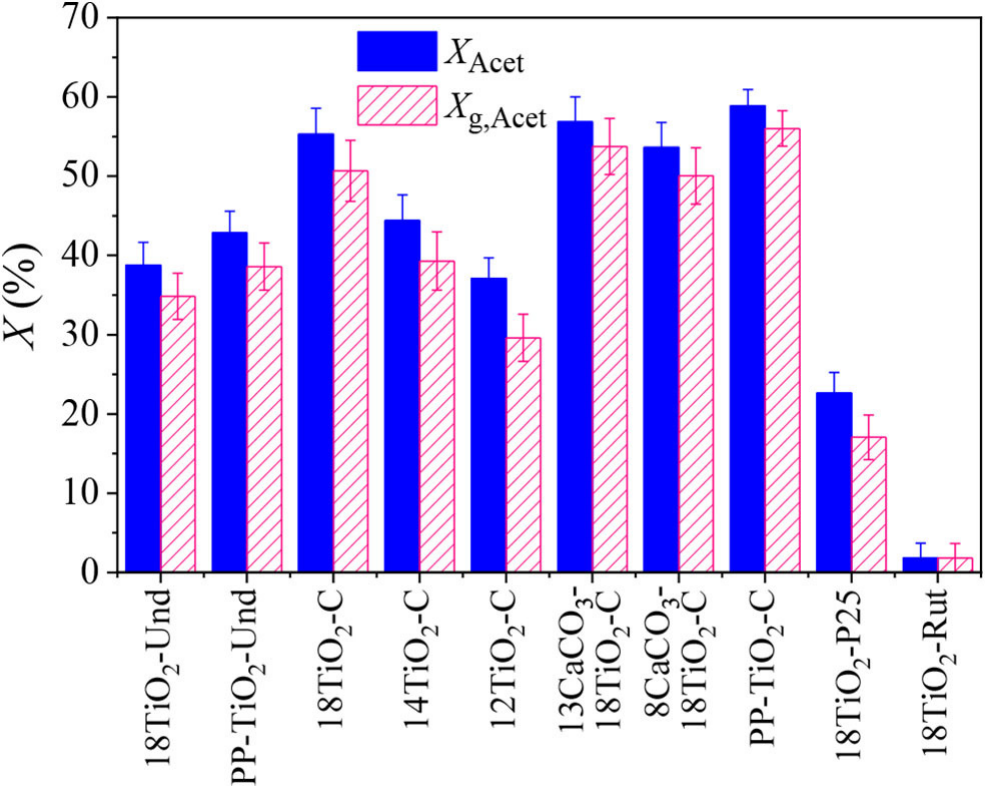
Acetaldehyde and global conversion for indoor like experiments and for different photocatalytic paints and pseudo-paints.

In the experiments with acetaldehyde, different pollutant conversions were achieved when the steady state was reached (Figure 5). Paints 18TiO_2_-C and PP-TiO_2_-C presented the best depollution capability. When the amount of carbon-doped TiO_2_ was decreased in the formulations of paints 14TiO_2_-C and 12TiO_2_-C, a reduction in acetaldehyde and global conversion was obtained. This is because the decreasing TiO_2_ and increasing CaCO_3_ amounts in the paint formulation results in a decrease in photocatalytic active area. On the other hand, paint 18TiO_2_-Und showed an acetaldehyde conversion similar to paint 12TiO_2_-C, although the paint with undoped TiO_2_ presented a slightly higher global conversion. It should be noticed that the 18TiO_2_-Und paint has presented photocatalytic activity under these reaction conditions because the fluorescent visible lamps emit small radiation peaks in the UV region (Supplementary Figure 1 of the Supplementary Material). Paint 18TiO_2_-P25 presented a reduced acetaldehyde conversion of 22.7%, although a higher conversion of 72.7% was found when this photocatalyst was tested alone, i.e., without any paint component, under the same operating conditions. As expected, paint 18TiO_2_-Rut showed no pollutant conversion.

Although the undoped and carbon-doped photocatalysts present the same band gap energy, it has been reported that the presence of carbonaceous species can increase the adsorption of pollutants and promote charge separation (Nyamukamba et al., 2012; Tobaldi et al., 2015; Khalid et al., 2017). This is a feasible explanation for the observed differences in acetaldehyde conversion of paints 18TiO_2_-C and 18TiO_2_-Und.

The pseudo-paints present about 4% higher acetaldehyde conversion than the photocatalytic paints. This effect can be attributed to the fact that, in the pseudo-paints, the amount of TiO_2_ is perceptually higher than in the other paints (Table 1), thus increasing the photocatalytic active area in the coating and the pollutants conversion. On the other hand, no effect on the photocatalytic performance could be observed when the amount of CaCO_3_ was reduced in paints 13CaCO_3_-18TiO_2_-C and 8CaCO_3_-18TiO_2_-C and maintaining constant the TiO_2_-C amount. However, varying the percentage of CaCO_3_ as an extender could result in the detriment of the physical properties of the paint, such as weatherability, gloss reduction, rheology, sedimentation and cracking, among others (Dörr and Holzinger, 1990).

**Figure 6:**
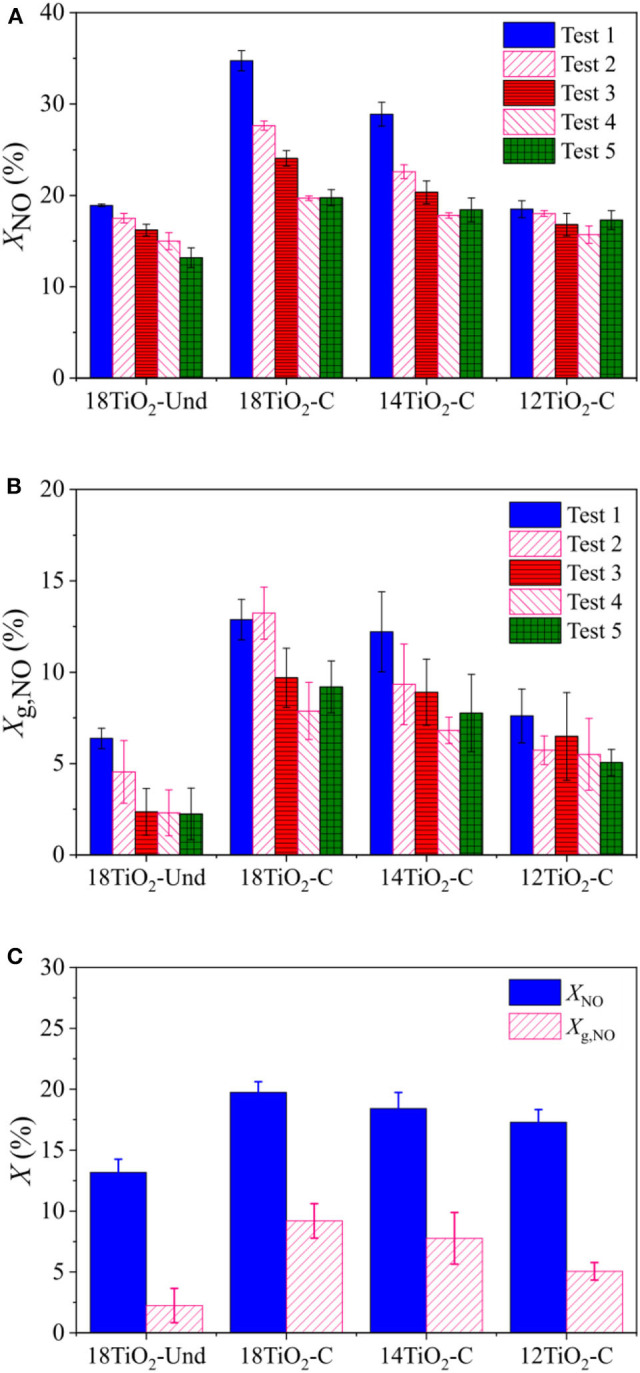
Conversion for outdoor like experiments and for different photocatalytic paints: **(A)** Subsequent tests for NO, **(B)** Subsequent tests for global conversion and **(C)** NO and global conversion for Test 5.

Figures 6A,B show the different NO and global (NO_*x*_) conversion capabilities under UV light for the diverse paint formulations and for the consecutive reaction tests. Similar to the indoor-like experiments, the paint 18TiO_2_-C presented the best conversion capability, followed by paints 14TiO_2_-C and 12TiO_2_-C. The paint 18TiO_2_-Und exhibited a NO conversion similar to paint 12TiO_2_-C, but the global NO_*x*_ conversion, contrary to the indoor-like experiments, is much lower. In general, the difference in NO and global conversions is very significant for all samples, which is due to the high formation of NO_2_ during the photoreaction. Given that the toxicity of NO_2_ is higher than that of NO, it is necessary to increase the selectivity toward non-toxic products, e.g., nitrogen doping and gold nanoparticles deposition on TiO_2_ (Luna et al., 2019).

For the outdoor-like experiments, it was observed that, as the tests proceeded, conversion ability diminished progressively for the different paints formulations (Figures 6A,B). However, it should be noticed that the activity decline for paint 12TiO_2_-C is not as abrupt as for the rest of the samples with higher TiO_2_ content. Before test 5, vacuum was applied to the paint samples with the purpose of desorbing volatile intermediates from the surface and recovering part of the lost conversion ability. This goal was partially achieved for paint samples containing carbon-doped TiO_2_ (18TiO_2_-C, 14TiO_2_-C and 12TiO_2_-C). However, the initial conversion could not be recovered fully after this procedure. Most likely, this detriment in the conversion capacity of the pollutant could be due to the adsorption of non-volatile nitrates on the paint precluding the reaction of TiO_2_ with the NO (Hunger et al., 2009). Finally, Figure 6C compares NO and global conversions for test 5.

In the indoor-like experiments, low volatile secondary pollutants, such as formic acid, could also be formed on the photocatalytic surface, but they would be easily oxidized and released into the air as CO_2_. Thus, the loss of photocatalytic activity during the test procedure was not observed in this system (Salvadores et al., 2020a).

Comparing the indoor- and outdoor-like experimental systems, the conversions for the NO experiments are significantly lower than for the acetaldehyde ones; in addition, the formation of the reaction intermediate is more important despite the fact that UV radiation is employed. This is mainly the result of, first, the illumination conditions. The total irradiation level that actually reaches the photocatalytic-coated plate is almost eight times higher for the visible than for the UV lamps. Second is the reactor residence time. The flowrate for the NO degradation experiments is three times larger than for the acetaldehyde ones. This fact along with the difference in the volume of the reactors results in a residence time almost 10 times larger for the acetaldehyde system than for the NO. Third is the photocatalytic area. The exposed catalytic area is more than eight times bigger for the acetaldehyde experiments than for the NO degradation.

### Efficiencies Evaluation

To be able to compare the radiation absorption capability of the photocatalytic paints under indoor- and outdoor-like experiments, the radiation absorption efficiency was calculated (Manassero et al., 2013):

(8) $$\begin{array}{l} \eta_{a}=\frac{\sum_{\lambda }e_{\lambda }^{a}}{\sum_{\lambda }\frac{E_{\lambda }}{E_{Total}}q_{w}T_{w,\lambda }}=\frac{\sum_{\lambda }e_{\lambda }^{a}}{\sum_{\lambda }\left(q_{w,\lambda }T_{w,\lambda }\right)} \end{array}$$

where *q*_*w*_ (Einstein/cm^2^/s) is the total incident radiation flux at the photoreactor window, *T*_*w*,λ_ is the spectral reactor window transmittance, *E*_λ_/*E*_*Total*_ is the spectral emission distribution of the lamp (Supplementary Figure 1 of the Supplementary Material), $e_{\lambda }^{a}$ (Einstein/cm^2^/s) is the LSRPA, and λ is the wavelength that can take values between 300 and 800 nm for the visible radiation source and from 300 to 450 nm for the UV radiation lamps. The denominator of Equation 8 is the radiation flux emitted by the lamps that reaches the photocatalytic paint surface, and the numerator is defined as:

(9) $$\begin{array}{l} e_{\lambda }^{a}=q_{w,\lambda }T_{w,\lambda }A_{TiO_{2},\lambda }\left(1+F_{q,\lambda }\right) \end{array}$$

where *A*_*TiO*2,λ_ = *A*_*TiO*2,*norm*,λ_ × *Weigth*/*Area* is the radiation absorption fraction of the TiO_2_ in the paint matrix calculated with the paint specific load of the reactor samples, and *F*_*q*,λ_ is the fraction of radiation that can cross the photocatalytic paint and the acrylic support (Salvadores et al., 2016). In the experiment where only one side of the photoreactor is illuminated, i.e., the outdoor-like experiments, *F*_*q*,λ_ is equal to zero. But when the photoreactor is illuminated from both sides, i.e., the indoor-like experiments, this term includes the light fraction coming from the back of the paint film where the $e_{\lambda }^{a}$ is being evaluated. *F*_*q*,λ_ can be calculated performing a radiative flux balance in the paint deposited on both sides of the acrylic plate (Ballari et al., 2016):

(10) $$\begin{array}{l} F_{q,\lambda }=\frac{R_{acr,\lambda }T_{paint,\lambda }-R_{acr,\lambda }^{2}R_{paint,\lambda }T_{paint,\lambda }+T_{acr,\lambda }T_{paint,\lambda }+T_{acr,\lambda }^{2}R_{paint,\lambda }T_{paint,\lambda }}{1-2R_{acr,\lambda }R_{paint,\lambda }+R_{acr,\lambda }^{2}R_{paint,\lambda }^{2}-T_{acr,\lambda }^{2}R_{paint,\lambda }^{2}} \end{array}$$

**Figure 7:**
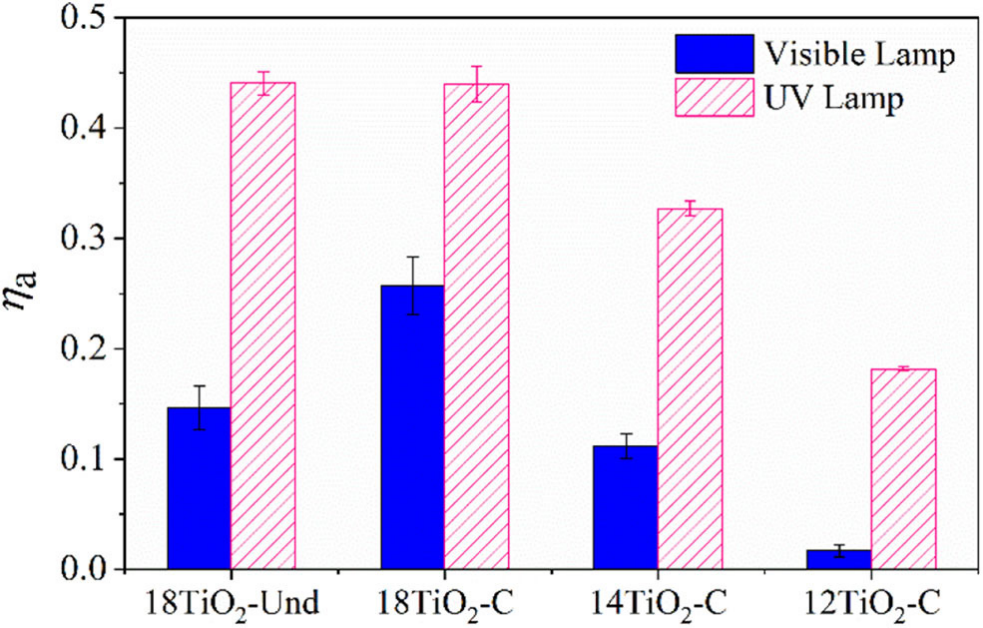
Radiation absorption efficiencies for the photocatalytic paints under visible and UV lights.

Figure 7 shows the radiation absorption efficiencies for the photocatalytic paints under UV and visible radiation sources. It can be seen that paints 18TiO_2_-Und and 18TiO_2_-C present almost the same efficiency for the UV lights. Nevertheless, the efficiency of paint 18TiO_2_-Und under visible illumination is significantly lower than the 18TiO_2_-C one. This is due to the fact that a visible-light carbonaceous sensitizer is present in the TiO_2_-C photocatalyst (Arimi et al., 2019). On the other hand, as the percentage of carbon-doped TiO_2_ in the paint formulation decreases, both the UV, and the visible light efficiencies decrease.

In order to be able to compare the depollution performance of the formulated paints under different operating and illumination conditions, the photonic, and the quantum efficiencies were calculated (Ballari et al., 2016). The PE was computed according to:

(11) $$\begin{array}{l} \eta_{p}=\frac{{\langle r\rangle }_{R}}{\sum_{\lambda }\left(q_{w,\lambda }T_{w,\lambda }\right)} \end{array}$$

And the QE was calculated as:

(12) $$\begin{array}{l} \eta_{q}=\frac{{\langle r\rangle }_{R}}{\sum_{\lambda }e_{\lambda }^{a}} \end{array}$$

where the numerators of Equations 11 and 12 represent the average reaction rate (mol/cm^2^/s) for the model pollutant “y” in the reactor volume, defined as:

(13) $$\begin{array}{l} {\langle r_{y}\rangle }_{R}=\frac{Q\left(C_{y,in}-C_{y,out}\right)}{Area} \end{array}$$

or the average global reaction rate considering the formation of the secondary pollutant “z”:

(14) $$\begin{array}{l} {\langle r_{g,y}\rangle }_{R}=\frac{Q\left(C_{y,in}-C_{y,out}-C_{z,out}\right)}{Area} \end{array}$$

In Equations 13 and 14, *Q* (cm^3^/s) is the gas flowrate, and *Area* (cm^2^) is the photocatalytic plate area exposed to radiation. According to the calculations (see Supplementary Material), both reacting systems are free from internal mass transfer limitations. On the other hand, the acetaldehyde oxidation is free from external mass transfer limitations, while the calculated values for NO degradation could indicate that there is a mild mass transfer limitation in part of the reactor. It has to be mentioned that the values used for calculations of the NO/NO_*x*_ experiments correspond to test 5.

**Figure 8:**
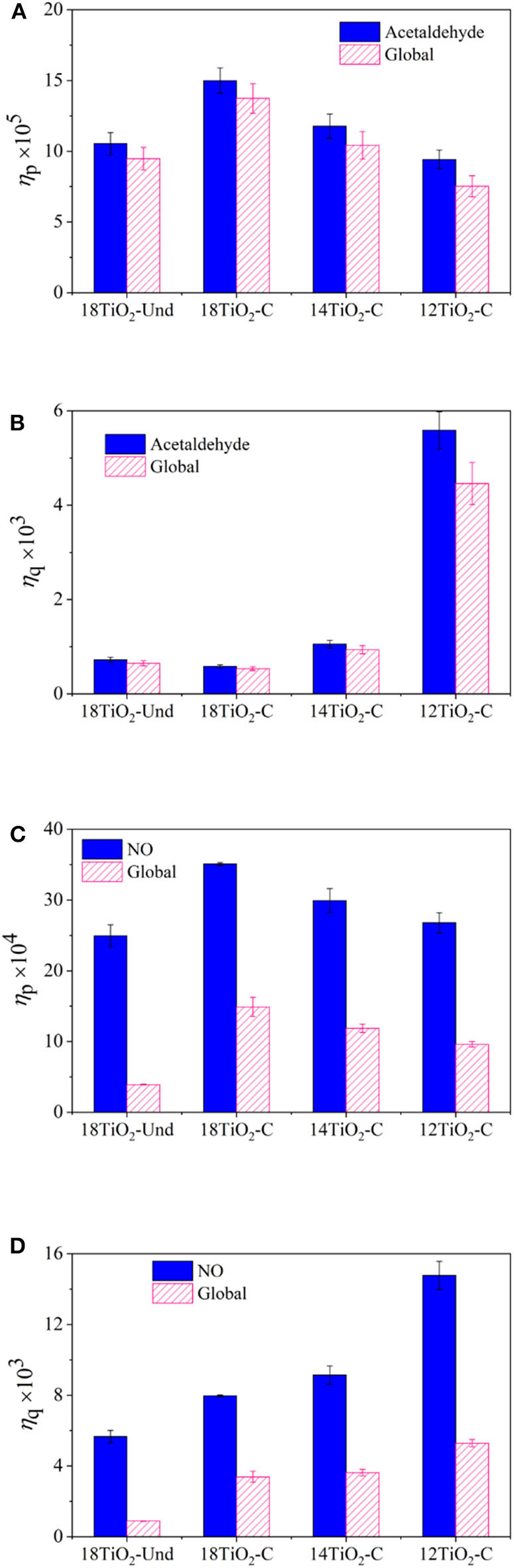
Efficiencies of the elaborated paints for main pollutant and global reaction rates: **(A)** PE for acetaldehyde reaction, **(B)** QE for acetaldehyde reaction, **(C)** PE for NO reaction and **(D)** QE for NO reaction.

Figures 8A,B show the photonic and quantum efficiencies, respectively, of the different paints under visible light conditions for the acetaldehyde and global reaction rates. Paint 18TiO_2_-C shows higher PE than paint 18TiO_2_-Und (Figure 8A). This indicates that paint 18TiO_2_-C makes better use of the incident light to activate the process. In addition, as the percentage of photocatalyst decreases, the PE declines. This is because decreasing the amount of TiO_2_ on the paint decreases the reaction rate, but the incident radiation flux remains constant in the denominator of Equation 11. On the contrary, a great increase in the QE is observed when the TiO_2_ amount is reduced (Figure 8B). In this case, both the denominator and the numerator in Equation 12 change, but the reaction rate decreases less than the radiation absorption rate. This can be explained by the fact that 12TiO_2_-C paint presents a higher Ti/O ratio at surface and lower particle agglomeration than the other formulations (Supplementary Table 2 of the Supplementary Material). Therefore, paint 12TiO_2_-C formulation makes better use of the effectively absorbed radiation to carry out the photoreaction.

For the UV illumination conditions, Figures 8C,D show the efficiencies of the photocatalytic paints for the NO and global oxidation. For this system, a similar trend as shown for the visible light illumination condition was obtained for the PE (Figure 8C) and QE (Figure 8D). It should be noticed that, in this case, all paints, but specially paint 18TiO_2_-Und formulation, exhibit a very low global efficiency in comparison with the NO photoreaction. This is because, in the NO oxidation experiments, high concentrations of NO_2_ were found causing the global average reaction rate to decline very much (see Equation 14). Even though acetaldehyde degradation experiments presented higher pollutant conversions, the higher PE and QE of the NO/NOx system is an indication of the good performance of TiO_2_ under UV light. Although the photonic efficiencies for the experiments under visible light and the ones under UV light differ by one order of magnitude, the results obtained are in good agreement with the values found in the literature for visible light (Tryba et al., 2014) and UV light (Ângelo et al., 2014; Monteiro et al., 2014, 2015).

## Conclusions

Different commercially available TiO_2_ powders were employed in the development of photocatalytic paint formulations. These paints were tested to study the degradation of a typical indoor VOC contaminant (acetaldehyde) under visible radiation and of an outdoor inorganic pollutant (NO) under UV radiation and to determine which paint presents the best decontamination performance through the methodology of efficiencies calculation. It was concluded that all the paints that contain carbon-doped TiO_2_ could degrade acetaldehyde and NO in gas phase, being the paint with the highest amount of photocatalyst (paint 18TiO_2_-C) and the best one in terms of conversion capability. In addition, the paint with undoped TiO_2_ shows good conversion in both systems, which, for indoor-like experiments, is due to small UV peaks emitted by the visible fluorescent lamps. The pseudo-paints showed a higher VOC conversion. Indeed, this effect was due to the higher photocatalyst percentage content in these pseudo-paints compared to the photocatalytic paints. When the TiO_2_-C amount was maintained constant while decreasing the CaCO_3_ amount in the paints, no effect on the pollutant conversion could be observed. Nevertheless, it should be considered that the calcium carbonate plays a fundamental role in the paint functionality. On the other hand, the NO and NO_*x*_ global conversion ability of the elaborated paints drops as the coatings are employed for several consecutive tests. This behavior could take place due to the adsorption of nitrates, the final product of the photoreaction on the paint surface. For the indoor-like experiments, this decline on the conversion ability was not detected.

The optical properties of the TiO_2_ in the paint coatings were determined to calculate the radiation absorption, photonic, and quantum efficiencies. The paint with the maximum amount of carbon-doped TiO_2_ (paint 18TiO_2_-C) presented the highest radiation absorption and photonic efficiency for both contaminants. In contrast, the paint with a lower amount of carbon-doped TiO_2_ (paint 12TiO_2_-C) showed the highest quantum efficiency, becoming the optimal formulation in terms of energy use. Throughout this work, it has been shown that different photocatalytic paint formulations can be a feasible technology for both reducing indoor and outdoor air pollution.

## Data Availability Statement

The raw data supporting the conclusions of this article will be made available by the authors, without undue reservation.

## Author Contributions

FS, MR, OA, KK, and MB designed the study and analyzed all the data. FS and MR performed the experiments and data collection. FS, OA, and MB made theoretical calculations. FS and MB wrote the main manuscript text and prepared the figures and tables. OA, KK, and MB obtained funding for the research. All authors reviewed the manuscript, made amendments, and contributed with their expertise.

## Conflict of Interest

The authors declare that the research was conducted in the absence of any commercial or financial relationships that could be construed as a potential conflict of interest. The handling editor declared a past co-authorship with one of the authors OA.

## Abbreviations

APS: average particle size
BET: Brunauer–Emmett–Teller
FID: flame ionization detector
ISO: International Standard Organization
LSRPA: local superficial rate of photon absorption
NO_*x*_: nitrogen oxides
PCO: photocatalytic oxidation
PE: photonic efficiency
PP: pseudo-paint
QE: quantum efficiency
Rut: rutile
SEM: scanning electron microscope
Und: undoped
UV: ultraviolet
VOC: volatile organic compound
w/w: weight/weight.
