# Supplementary material for: Efficiencies Evaluation of Photocatalytic Paints Under Indoor and Outdoor Air Conditions
Source: Front Chem. 2020 Oct 23;8:551710. doi: 10.3389/fchem.2020.551710 (PMC7650231; doi:10.3389/fchem.2020.551710)
Supplement: Supplementary file 1 [file Data_Sheet_1.docx]

Supplementary Material

# Internal and external mass transfer limitations

To know if the average reaction rate is free from internal and external mass transfer limitations, the external and internal mass transfer modulus for the diverse paints and reactors is calculated.

The external mass transfer modulus can be calculated as (Ballari et al., 2016):

$MTM_{\text{ext,y}}\text{=}\left\langle r_{\text{y}}\right\rangle_{\text{R}}/\left[k_{\text{s}}\left\langle C_{\text{y}}\right\rangle_{\text{R}}\right]$ (ESM.1)

where $\left\langle C_{\text{y}}\right\rangle_{\text{R}}$ (mol/cm^3^) is the average concentration of the pollutant in the reactor and *k*_s_ (cm/s) is the external mass transfer coefficient that can be calculated from the Sherwood number:

$Sh\text{=}k_{\text{s}}L_{\text{c}}/D_{\text{y-air}}$ (ESM.2)

Here *L*_c_ (cm) is the system characteristic length and equal to the hydraulic diameter of the reactor, and *D*_y-air_ (cm^2^/s) is the molecular diffusion coefficient of the pollutant in air. The Sherwood number could be taken as 5 for slits (Shah and London, 1974).

On the other hand, the internal mass transfer modulus is:

$MTM_{\text{int,y}}\text{=}\left(1/H\right)\left\langle r_{\text{y}}\right\rangle_{\text{R}}e^{2}/\left(D_{\text{e}}C_{\text{s,y}}\right)$ (ESM.3)

where *H* (cm) is the thickness of the reactor, *e* (cm) is the thickness of the paint deposited on the plate, *C*_s,y_ (mol/cm^3^) is the pollutant concentration on the paint film surface, and *D*_e_ (cm^2^/s) is the effective diffusion coefficient which is a function of *D*_y-air_, the Knudsen diffusion coefficient and the porosity and tortuosity of the paint film. The thickness of the paint was measured from the SEM images resulting in an average of 1.2×10^-3^ cm.

Table ESM1 shows the values of the external and internal modulus for both indoor and outdoor like reactors and pollutants. If the modulus is lower than 1, it means that the reaction rate is slower than the mass transfer, which is true for both systems. It should be noticed that the highest external mass transfer modulus for the outdoor like experiments is 0.26, which could indicate the existence of some mass transfer limitation during the NO degradation in some part of the reactor.

# Supplementary Figures and Tables

## Supplementary Figures





**Supplementary Figure 1.** Spectral emission distribution of visible and UV lamps.


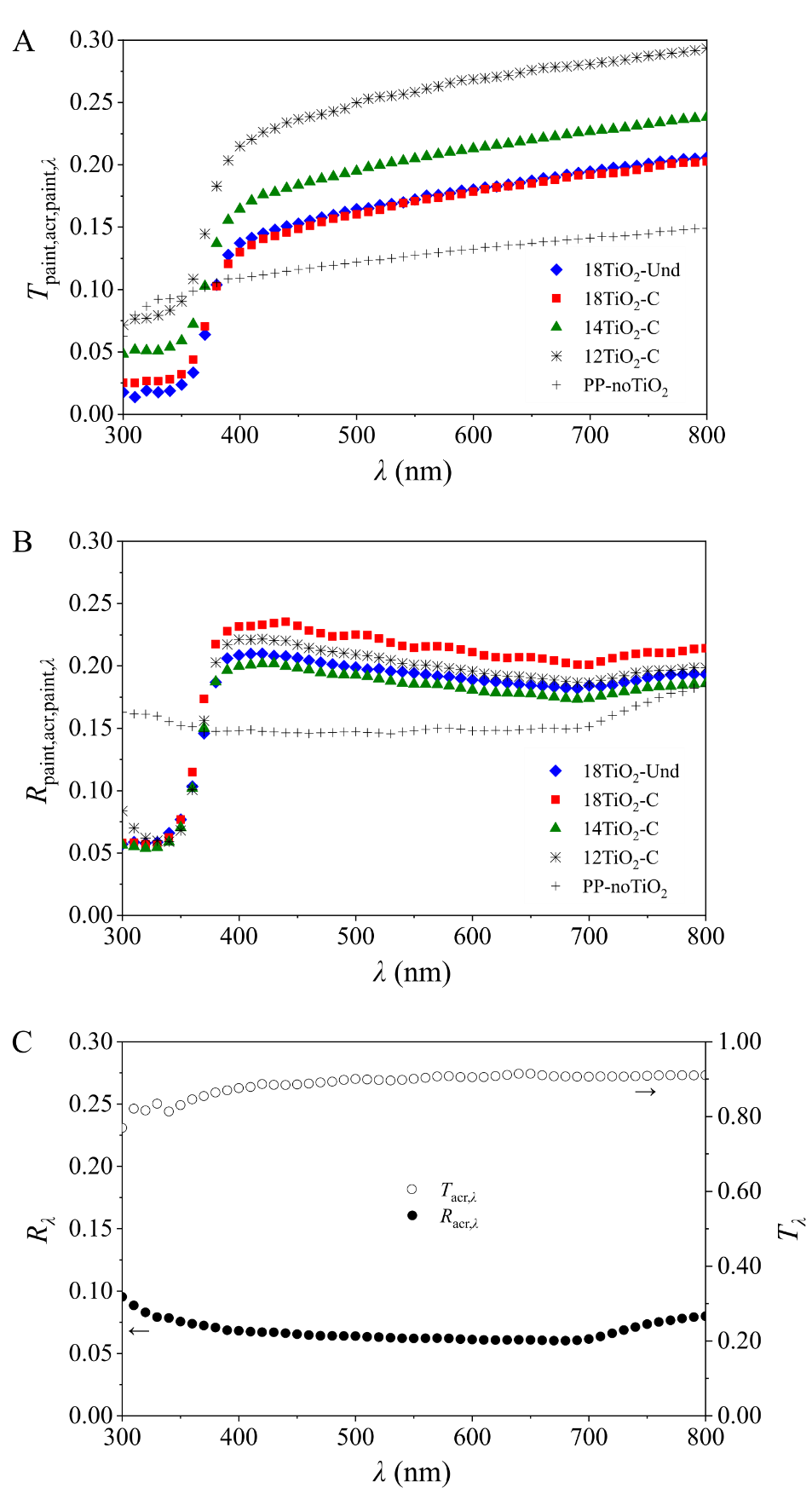


**Supplementary Figure 2.** Measured spectral diffuse: (A) transmittance of coated acrylic plates, (B) reflectance of coated acrylic, (C) transmittance and reflectance of acrylic plate without paint coating.

**Supplementary Figure 3.** X-ray photoelectron (XPS) spectra and deconvoluted peaks of: (A) Ti 2p for 18TiO_2_-C before irradiation, (B) O 1s for 18TiO_2_-C before irradiation, (C) Ti 2p for 18TiO_2_-C after irradiation, (D) O1s for 18TiO_2_-C after irradiation, (C) Ti 2p for 12TiO_2_-C after irradiation, (D) O 1s for 12TiO_2_-C after irradiation. The analysed bands were 2p for the Ti and 1s for O and for C. The reference was C 1s, and the spectra were corrected at 284.6 eV. The measured signal in all the samples was 458.9 ± 0.2 eV compatible with TiO_2_. The oxygen signal corresponding to the photocatalyst is masked due to the presence of the oxygens of the resin and CaCO_3_, thus total O 1s is used in the Ti/O ratio.

## Supplementary Tables

**Supplementary Table 1** External and internal mass transfer modulus

| Paint | Indoor like experiments | | Outdoor like experiments | |
| --- | --- | --- | --- | --- |
|  | External  modulus ×10^2^ | Internal  modulus ×10^5^ | External  modulus ×10^2^ | Internal  modulus ×10^5^ |
| 18TiO_2_-Und | 1.1 | 1.9 | 19.5 | 5.0 |
| 18TiO_2_-N | 0.1 | 0.2 | 0.2 | 0.1 |
| 18TiO_2_-C | 1.8 | 3.0 | 26.3 | 6.8 |
| 14TiO_2_-C | 1.3 | 2.2 | 23.5 | 6.1 |
| 12TiO_2_-C | 1.1 | 1.8 | 20.5 | 5.3 |

**Supplementary Table 2** X-ray photoelectron spectroscopy (XPS) measurements of surface atomic composition (10 nm deep).

| Paint coating | O (%) | Ti (%) | Ti/O ratio |
| --- | --- | --- | --- |
| 18TiO_2_-C before irradiation | 13.0 | 0.1 | 0.011 |
| 18TiO_2_-C after irradiation | 12.7 | 0.2 | 0.015 |
| 12TiO_2_-C after irradiation | 12.1 | 0.2 | 0.019 |

# References

Ballari, M. M., Carballada, J., Minen, R. I., Salvadores, F., Brouwers, H. J. H., Alfano, O. M., et al. (2016). Visible light TiO_2_ photocatalysts assessment for air decontamination. *Process Saf. Environ. Prot.* 101, 124–133. doi:10.1016/j.psep.2015.08.003.

Shah, R. K., and London, A. L. (1974). Thermal boundary conditions and some solutions for laminar duct flow forced convection. *J. Heat Transfer* 96, 159–165. doi:10.1115/1.3450158.
